# Hydraulic Retention Time as an Operational Tool for the Production of Short-Chain Carboxylates via Anaerobic Fermentation of Carbohydrate-Rich Waste

**DOI:** 10.3390/molecules28186635

**Published:** 2023-09-15

**Authors:** Kaoutar Aboudi, Silvia Greses, Cristina González-Fernández

**Affiliations:** 1 Biotechnological Processes Unit, IMDEA Energy, Avda. Ramón de la Sagra 3, 28935 Madrid, Spainsilvia.greses@imdea.org (S.G.); 2 Department of Chemical Engineering and Environmental Technology, School of Industrial Engineering, University of Valladolid, Dr. Mergelina, s/n, 47002 Valladolid, Spain; 3 Institute of Sustainable Processes, Dr. Mergelina, s/n, 47002 Valladolid, Spain

**Keywords:** anaerobic fermentation, hydraulic retention time, short-chain fatty acids, chain elongation, caproic acid

## Abstract

The carboxylate platform is a sustainable and cost-effective way to valorize wastes into biochemicals that replace those of fossil origin. Short-chain fatty acids (SCFAs) are intermediates generated during anaerobic fermentation (AF) and are considered high-value-added biochemicals among carboxylates. This investigation aimed to produce SCFAs through the AF of sugar beet molasses at 25 °C and semi-continuous feeding mode in completely stirred tank reactors. A particular focus was devoted to the role of hydraulic retention time (HRT) variation in SCFAs production and distribution profile. The highest SCFAs concentration (44.1 ± 2.3 gCOD/L) was reached at the HRT of 30 days. Caproic acid accounted for 32.5–35.5% (COD-concentration basis) at the long HRTs of 20 and 30 days due to the carbon chain elongation of shorter carboxylic acids. The findings of this study proved that HRT could be used to steer the anaerobic process toward the targeted SCFAs for specific uses. Furthermore, the successful operation at low-temperature conditions (i.e., 25 °C) makes the process economically promising.

## 1. Introduction

The carboxylate platform is a cost-effective way to valorize a wide range of residual biomass (e.g., agricultural wastes and by-products, animal manure, urban and food wastes, sewage sludge, etc.) into biofuels and biochemicals able to replace petrochemicals at some extent. Carboxylates are short-chain fatty acids (SCFAs) with industrial demand that might be produced via waste anaerobic fermentation (AF). AF can be regarded as an altered conventional anaerobic digestion (AD) by inhibiting methanogenic archaea to promote the acidogenesis step, leading to SCFAs accumulation [1,2]. Compared to biomethane, SCFAs have higher economic value and a wider range of applications through biological (i.e., oils, polyhydroxyalkanoates) or chemical conversion (i.e., esters, polymers) for their use in pharmaceutical, cosmetic, or food industries [3].

AF occurs through a series of biochemical reactions in which acidogenic bacteria ferment the hydrolyzed wastes into SCFAs (mainly acetate C2, propionate C3, and butyrate C4), alcohols, together with hydrogen and carbon dioxide [4]. Under suitable operational conditions, the microbial consortia could further produce longer fatty acids (i.e., caproate C6 and caprylate C8) through the carbon chain elongation (CCE) process. CCE of SCFAs occurs in an energy-rich environment and reducing agents [5,6].

Like conventional AD, various factors influence AF, including temperature, pH, hydraulic/solid retention time (HRT/SRT), organic loading rate (OLR), etc. Concerning pH, slightly acidic conditions (5.5–6.5) have been determined as the most suitable range for SCFAs production, especially at low-temperature conditions (i.e., 25 °C) [1,7].

HRT, equal to SRT in completely stirred tank reactors (CSTRs), refers to the average time microorganisms are in contact with the substrate in an anaerobic reactor. In this sense, an appropriate HRT must be set up to avoid the washout of microorganisms. Whereas low HRTs imply a low residence time of microbes inside reactors, high HRTs allow microbes to have enough time to grow and degrade organic matter more efficiently. Considering that the growth rate of methanogenic archaea is lower than that of acidogenic bacteria [8], HRT control can be used to force the washout of methanogens from the reactor, enabling the selection of the most suitable populations for SCFAs accumulation. It cannot be neglected that each AF process could have a different response to HRT variability according to the nature of the substrate used and the implemented operational conditions (i.e., temperature, pH, reactor configuration, etc.) [8,9]. Indeed, AF for SCFAs production has been intensively investigated using different waste streams (mainly food waste and sewage sludge) and process configurations to either increase production or obtain a specific distribution profile. Proof thereof is the wide number of reviews addressing the AF technology [10,11,12,13].

This investigation selected carbohydrate-rich waste (sugar beet molasses, SBM) as a substrate to be valorized via AF to produce SCFAs. SBM is considered a valuable by-product of the sugar production industry. The most common use of this waste is as a supplement for animal feed. However, previous renewable energy directives, such as the Renewable Energy Directive 2009/28/EC [14], classified molasses as an advanced biofuel and bio-based chemical feedstock. Since SBM could be alternatively valorized as SCFAs and contribute to greening the chemical industry, this waste was considered a suitable feedstock for AF. Furthermore, since the HRT implemented in the reactors ultimately affects the production yields and operational costs, the novelty of this investigation lies in the identification of the suitable HRT that can be applied without compromising the yields of this waste into SCFAs at low temperatures, as well as the targeted individual SCFAs. In addition, perspectives on using SBM for SCFAs versus the current use as an animal feed supplement were addressed.

## 2. Results and Discussion

### 2.1. Performance of the Hydrolytic and Acidogenic Activities in the AF of SBM

**Figure 1:**
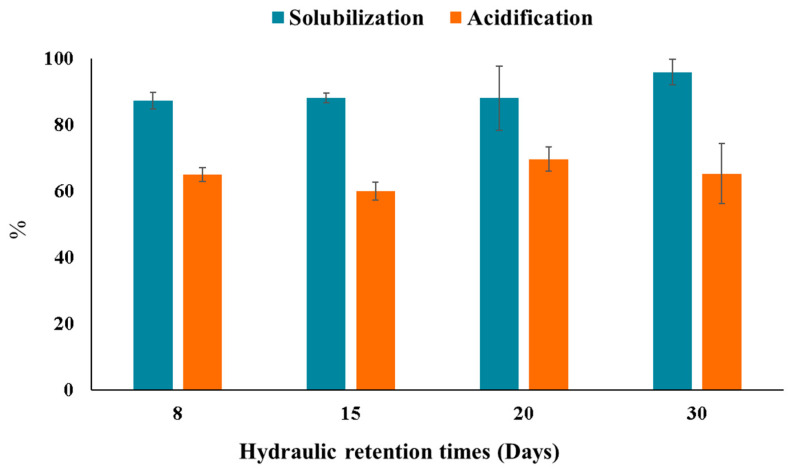
Solubilization and acidification degrees achieved in the SBM-AF at different HRTs.

Figure 1 shows the solubilization and acidification degrees of SBM. SBM was expected to be a readily fermentable substrate (SCOD/TCOD ratio of 94.3%). Therefore, the solubilization degree, which assesses the hydrolytic activity in the reactors, was high at all the HRTs tested, reaching values ranging from 85 to 95%. The highest solubilization performance was observed in the reactors operating at HRTs of 30 and 20 days (95.8 ± 2.5 and 90.6 ± 1.4%, respectively). In CSTR, long HRTs (i.e., low flow rates of the feeding) enable longer reaction times for microorganisms to solubilize the organic material of substrates. When using complex substrates for AF, the hydrolysis step often takes a long time and is regarded as the rate-limiting stage to obtain soluble monomers, such as amino acid, long-chain fatty acid, monosaccharide, etc. [15]. In contrast, for highly soluble substrates, such as SBM, the acidogenesis step gains relevance because this process occurs faster than with complex substrates. This fact is the main reason of the high SCOD percentage reached regardless of the HRT evaluated.

It should be considered that acidogenesis is a fast step and, thereby, a high content of metabolites can be released altering the medium properties. pH is one of the parameters that most frequently gets affected by this release, decreasing its value towards acid values. Since methanogens are the most sensitive microorganisms in the context of low pHs, such a circumstance can be considered as an advantage in AF because SCFAs would be accumulated and not consumed for methane production [16].

Nevertheless, an excessive pH drop can also lead to an acidogenesis inhibition, resulting in a SCFAs concentration decrease. Previous studies reported that a decrease in pH values below 5.5 might result in the accumulation of primary fermentation products such as lactic acid and/or ethanol, which hinders the involved pathways for SCFAs generation [3,17]. Thus, AF process stability is strictly related to the pH values in the culture broth, affecting the SCFAs concentration achieved [17,18]. In the present research, pH fluctuations were higher at low HRTs than at the longest ones (Supplementary Material). At 20 and 30 days, pH dropped below the desired limit (5.5–6.5) only at the start-up period, while fluctuations were more pronounced for short HRTs. In this regard, the high consumption of the alkali reagent (i.e., NaOH) for the pH adjustment in reactors operating at short HRTs throughout the experiments may increase the operational cost, particularly at a large scale. In contrast, proper pH control without excessive reagent consumption allowed working at prolonged HRTs (i.e., 20–30 days), avoiding acidogenic system instability. The same pH-related issue was reported by Jankowska et al. [18] when working at different HRT. The rapid pH decrease in reactors fed with sugar-rich substrates is a common feature given the fast hydrolysis rates of carbohydrates compared to other macromolecules such as proteins and lipids [19]. In this sense, HRT selection (or any other operational parameter) should be selected not only in terms of SCFAs concentrations and yields but also in terms of process stability.

Concerning the acidification degree, all HRTs resulted in high values (60–70%, Figure 1) comparable to other studies dealing with carbohydrate-rich substrates [2,17]. Greses et al. [20] obtained a high acidification degree (83%) for AF of a carbohydrate-rich waste (79% of carbohydrates) by increasing the process temperature (55 °C). Nevertheless, the temperature increase involved a high-energy input that did not offset 13 percentual points of acidification enhancement. Overall, these results evidenced that AF of SBM can reach stable and high acidogenic performance regardless of the HRT when the values varied between 8 and 30 days.

### 2.2. Evaluation of the AF in Terms of SCFAs Production

**Figure 2:**
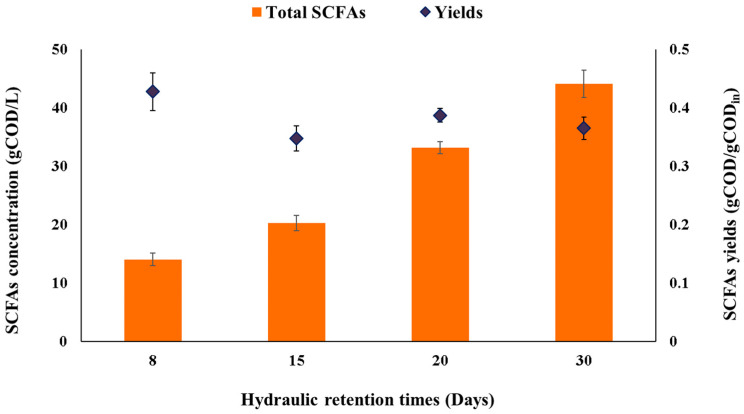
Total SCFAs productions and the obtained yields from AF of SBM at different HRTs.

As shown in Figure 2, increasing the HRT concomitantly increased the total concentration of SCFAs produced. Reactors operating at HRTs of 30 and 20 days showed the highest SCFAs accumulations, namely 44.1 ± 2.3 g COD/L (27.6 ± 1.6 g SCFAs/L) and 33.2 ± 1.1 g COD/L (20.4 ± 0.5 g SCFAs/L), respectively, whereas operating at short HRTs led to SCFAs concentrations of 21.2 ± 1.3 g COD/L (11.5 ± 0.6 g SCFAs/L) and 14.1 ± 1.1 g COD/L (8.5 ± 0.6 g SCFAs/L) for HRTs of 15 and 8 days, respectively (Figure 2 and Table 1).

These concentrations represented high values compared with previous investigations using carbohydrate-rich waste as feedstock. For instance, Onodera et al. [21] reported SCFAs concentrations of 15.6 and 25.9 g COD/L at HRTs of 13.1 and 18.2 days, respectively, when performing AF of diluted cane molasses (with high content of sucrose) at 35 °C. The high concentrations in the present research at similar HRTs revealed that 25 °C was a more suitable temperature than 35 °C when feedstocks with high readily organic matter content are subjected to AF. Moreover, this fact is relevant when energy costs are considered. Likewise, Bolaji and Dionisi [3] found that for the mesophilic AF of carbohydrates-rich food waste, the highest SCFAs concentration of 19.4 g COD/L was obtained at 30 days of HRT. That value was comparably lower than the one attained herein (44.1 ± 2.3 g COD/L for HRT at 30 days), highlighting the optimum conditions implemented other than retention time. The positive effect of low AF temperature can be thus confirmed by comparing with previous studies since only those conducting AF of carbohydrate-rich waste at 25 °C reported such high SCFAs concentrations [2,22].

In a conventional AD process, high HRTs are commonly applied for methane-rich biogas production, while short HRTs have been shown to benefit hydrogen-rich biogas generation. This is mainly due to the slow growth rate of methanogens compared to hydrolytic/fermentative acidogens [8]. Specifically, HRT has been shown to affect the relative abundance of each microbial community [16,23]. The underlying reason for this is based on the fact that HRT determines the daily flow rate of substrate fed to the reactor, which affects the available time for microorganisms to degrade the organic material. Llamas et al. [16] reported that short HRTs led to a 75% decrease in SCFAs concentration (i.e., from 5.2 to 1.3 g COD/L at HRTs of 10 and 4 days, respectively) due to the high flow rate, which triggers the wash out of the hydrolytic/acidogenic microorganisms from the system.

Along with SCFAs concentrations, high SCFAs yields ranging from 0.35 ± 0.02 g SCFA/g COD_in_ (0.49 ± 0.05 g SCFA/g VS_in_) to 0.43 ± 0.03 g SCFA/g COD_in_ (0.66 ± 0.05 g SCFA/g VS_in_) were reached at all HRTs, with the highest value observed at 8 days of HRT (Figure 2 and Table 1). This could be due to the need to dilute the organic material content of the daily feedstock (inlet flow rate) to keep the OLR constant at 4 g COD/L d. The obtained SCFAs yields were in line with the values reported for carbohydrate-rich substrates. For instance, Greses et al. [2] obtained SCFAs yields of 0.43–0.46 g SCFA/g COD_in_ in AF of highly carbohydrate-rich vegetable waste (i.e., 75.7–80.4% of carbohydrates) at pH values around 5.6–5.8 and 25 °C. In the case of using a substrate with low carbohydrate content (i.e., vegetable wastes with 5.8% of carbohydrates), AF at 37 °C and pH values of 5.6–5.8, the SCFAs yield dropped to 0.21 g COD/g VS_in_ compared to 0.45 g COD/g VS_in_ from AF of a vegetable waste with a carbohydrate content of 45% [24]. Thereby, it can be suggested that as carbohydrate content in the feedstock decreases, the carboxylates yields also do. Conversely, AF of protein-rich substrates such as microalgae led to low SCFAs yields, as reported by Magdalena et al. [6], highlighting that high SCFAs yields are aligned with carbohydrate-rich substrates.

### 2.3. SCFAs Distribution Profiles upon Different HRT

**Figure 3:**
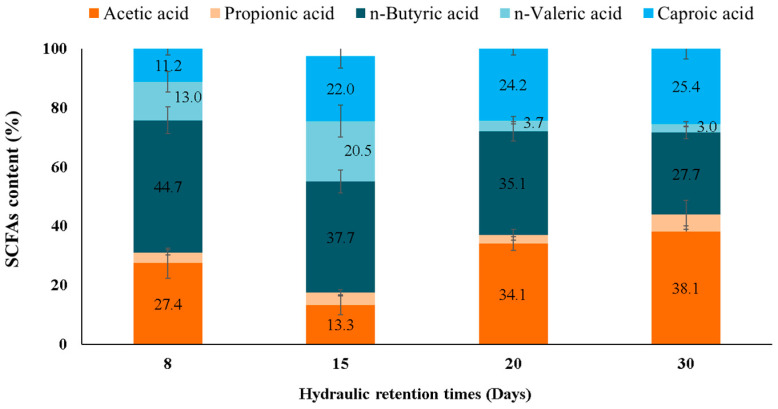
SCFAs distribution profile achieved in the SBM-AF at different HRTs.

The distribution profile of carboxylic acids is paramount in AF as it determines the possible end-uses of the harvested SCFAs. Figure 3 depicts SCFAs profile distribution according to HRT variation (expressed as SCFA/Total SCFAs × 100).

As a general trend, it can be observed that longer carbon chain carboxylates were favored at longer HRTs. This pattern was in agreement with other studies [2,25].

A similar SCFAs distribution was observed at the longest HRTs (30 and 20 days). Acetic acid (38.2 ± 2.3% and 34.1 ± 3.2%), butyric acid (27.7 ± 2.1% and 35.1 ± 3.2%), and caproic acid (25.4 ± 2.9% and 24.2 ± 1.2%) were the primary carboxylic acids at HRTs of 30 days and 20 days, respectively. At 15 days of HRT, butyric acid presence prevailed with values of 37.6 ± 4.5%, while valeric and caproic acids accounted for almost the same (20.5 ± 1.1% and 21.9 ± 1.8%, respectively). For the short HRT of 8 days, butyric acid made up 44.7 ± 3.7%, followed by acetic acid at 27.4 ± 4.1%, whereas valeric and caproic acids exhibited almost equal proportions (11.2–13.1%). Propionic acid proportions were negligible in all reactors.

As stated in the previous section, HRT variation not only affected microbial population abundance but it may affect as well the predominant pathway of each individual SCFA [8,23] and, therefore, the final concentrations. Indeed, high HRTs have been reported to provide higher concentrations of more reduced compounds (i.e., long molecules such as butyrate, valerate, and caproate) rather than the more oxidized ones (i.e., short molecules such as acetate) [25,26].

The AF of carbohydrate-rich substrates usually produces even-chain carboxylic acids, whereas protein-rich substrates result in odd-chain carboxylates [17,22]. The molasses used as feedstock in the present investigation is considered a sugar-rich substrate, which justified the prevalence of even-chain carboxylates (acetic, butyric, and caproic acids). In addition to the substrate type (e.g., prevailing in carbohydrates, proteins, or lipids), operational parameters such as temperature, HRT, hydrogen partial pressure (*p*H_2_), and inoculum type, among others, also affect the distribution of yielded SCFAs [8,11]. For instance, the effect of temperature on SCFAs production and profiles was studied by Greses et al. [27] in combination with the macromolecular composition of the organic material. Those researchers found that the temperature setup at 25 °C increased caproic acid content for carbohydrate-rich substrates, given that a low temperature hampers the conversion of long fatty acids into shorter ones.

Regarding *p*H_2_, in a conventional AD process aiming to produce methane, the *p*H_2_ must be low enough for a thermodynamically favored degradation of SCFAs into methane precursors [13]. For instance, Cazier et al. [28] reported that a high *p*H_2_ inhibited the acetogenesis stage in solid-state mesophilic AD and lowered methane production. Likewise, the syntrophic oxidation of alcohols and SCFAs greatly depends on H_2_. In this regard, the oxidation of SCFAs is only thermodynamically feasible at low *p*H_2_ values [28]. The syntrophic oxidation can only spontaneously occur when H_2_ consumption takes place, being the presence of H_2_-consumers a requirement [29]. Because the Gibbs free energy of SCFAs oxidation reactions are positive under standard conditions, the process becomes thermodynamically favorable when methanogens maintain *p*H_2_ lower than 10^−4^ atm [30]. In this investigation, the H_2_ production attained (Table 1) could be responsible of the syntrophic oxidation limitation. This fact would justify the prevalence of longer SCFAs (butyric and caproic acids) over acetic acid.

Metabolite production in open, mixed cultures processes does not usually occur via one single pathway, given the co-occurrence of several bioreactions.. This fact gains relevance when non-standard conditions are applied since the metabolic role of microorganisms can also be altered. In this regard, one of the main singularities presented herein was the high accumulation of caproic acid at long HRTs (Figure 1). While some authors described this phenomenon as a consequence of an acetogenesis inhibition [6], recent investigations determined CCE as main pathway for caproic production when AF was performed at acid pH and long HRTs [2,31]. CCE enables the conversion of acetic and/or butyric acid into caproic acid in presence of an electron donor (ED), namely lactic acid or ethanol. Both EDs are primary intermediate metabolites of AF, mostly promoted at a low pH value. Thus, the prevalence of caproic acid at long HRTs compared to the short ones was likely due to the availability of ethanol and/or lactate produced via the primary fermentation of SBM. Moreover, it should be taken into account that CCE has been described as a hydrogenogenic pathway [2]. As can be seen in Table 1, the observed pattern of SCFAs and the H_2_ production determined at long HRTs confirmed that caproic acid formation was likely attained via CCE.

It is essential to highlight that those EDs are normally added to the fermentation broth as an external supply to promote the CCE. For instance, Contreras-Dávila et al. [32] obtained 10.8 g COD/L of caproic acid from AF of food waste by adding lactate, and Roghair et al. [33] achieved a higher concentration of 51.6 g COD/L by adding ethanol to the anaerobic system. In contrast, in the present investigation, high caproic acid concentrations were attained without adding any external chemicals. During the steady state of all CSTRs, particularly those operating at long HRTs, lactic acid was not detected in the fermentation broth. However, when pH correction was necessary to avoid the drop below the threshold of 5.5, lactic acid was observed, although in small concentrations (0.10–0.35 g/L), corroborating the probable use of lactic acid as an ED when the CCE occurred at suitable pH values (i.e., 5.5–6.5). Previous studies found that process temperature also play an important role in the CCE since 25 °C seems to favor the process, while increasing fermentation temperatures to 55 °C may present a thermodynamic impediment [2,20]. These results showed that in situ CCE can be promoted by combining low temperatures (25 °C) with slightly acid pHs (5.5–6.0). Although ethanol and lactic acid exhibit similar Gibbs free energy as ED in CCE [34], it has been reported that lactic acid is preferred as ED [35]. This fact, along with the detection of lactic acid in the CSTR, might indicate that caproic acid accumulation was related to the use of lactic acid as ED for CCE.

CCE is a novel process gaining much attention recently due to the high economic value of the longest carboxylates in the material and chemical markets [4,33]. For instance, caproic acid is an increasingly growing biochemical platform due to its high economic value, which reached $3815/ton, compared to $600/ton for acetic acid [33]. Caproate can be used in various industrial applications. Some of those include the production of flavor compounds and antimicrobials [5] or biofuels such as diesel and jet fuels due to its similarities to gasoline in storage and transport [36].

Nevertheless, the present investigation produced a high concentration of caproic acid (e.g., 15.4 ± 1.5 g COD/L at HRT of 30 days) in situ without providing any external ED, reducing exogenous chemicals input in the fermenters and operating costs. Moreover, the high percentage of a specific fatty acid in the fermentation broth makes its separation easier for recovery. The high percentage and the fact that this acid exhibits high energy density and hydrophobicity enhance its insolubility and, therefore, its separation [36].

### 2.4. Organic Matter Removal (COD) in the AF upon the Different HRT Tested

COD removal in the CSTRs operated at different HRTs is depicted in Table 1. COD removal was higher (31–41%) at long HRTs than at the short HRT of 8 days (23%). This was aligned with previous AF studies. Bolaji and Dionisi [3] reported that for AF of carbohydrate-rich substrates, COD removal increased when increasing HRTs, with the highest COD removal of 35% at HRT of 30 days. In the present investigation, the observed COD removal values were partially attributed to the chain elongation process that was promoted at long HRT due to caproic acid formation at these retention times. Being caproic acid a hydrogenogenic process that releases hydrogen when lactic acid is involved in the elongation process [37], the gas release could be partially related to the high COD removal attained at long HRT. In this study, hydrogen was observed during AF of SBM as a co-product of SCFAs (Table 1). These hydrogen yields were lower than the reported in the literature from similar substrates where the experiments were directed toward hydrogen production rather than SCFAs, where favored microbial pathways were different [2,37].

In a complex biological process such as AF, the organic matter removal (e.g., COD) from the system could be attributable to several microbiological pathways than its conversion into SCFAs (e.g., CH_4_, H_2_, N, and P removal, etc.). In this study, the high COD removal attained without observing significant gas production suggested that some solubilized COD fractions were probably used by other microorganisms through different pathways. For instance, previous authors showed that adding a fermentation broth rich in SCFAs to a nutrient removal system significantly enhanced the process. This further supports the versatility of SCFAs as a high-value-added product in various applications [38].

Since the present research aimed to maximize SCFAs production by intervening only in applying different HRTs under specific temperature and pH conditions, determining which metabolic pathway was responsible for the COD consumption was out of the scope. However, it was highly relevant to know that long HRT diverted COD towards other pathways rather than the targeted COD conversion into SCFAs. In this way, when aiming at SCFAs accumulation, shorter HRTs were suggested to be more profitable since these HRTs resulted in the lowest COD consumption.

### 2.5. Economic Perspectives of the Results and Future Scope

Worldwide sugar beet production was around 270.16 million tons in 2021 [39]. Per each ton of sugar beet processed, around 40 kg of beet molasses are generated, producing about 10,8 million tons of global molasses [40]. Molasses are usually used as a supplement in animal nutrition [39] or in the production of fuels (i.e., bioethanol), as well as in the production of yeasts, vitamins, and other components for the food and beverages industries [39]. However, the low economic value of molasses as an animal feed supplement limits interest in this use. Furthermore, thick sugar juices generated during sugar production, starch, or glucose-based substrates are preferred feedstock in the fermentation industry rather than molasses [41]. In this context, producing other value-added products from molasses is promising and is currently attracting interest from the sugar industry stakeholders to find new outlets as a driver for the sector’s competitiveness [40]. The chemical characteristics of molasses make them an attractive substrate in biotechnological processes, such as anaerobic digestion to produce biogas or carboxylic acids in the anaerobic fermentation process [42,43,44]. The global value of molasses is about seven billion USD [41]. In the EU, beet molasses cost 125–152 $/tons in 2019 [41]. Considering the average price of 2 $/kg of molasses as animal feed, the revenues from the molasses used for this purpose in 2021 could be around $21,600 billion [41].

**Figure 4:**
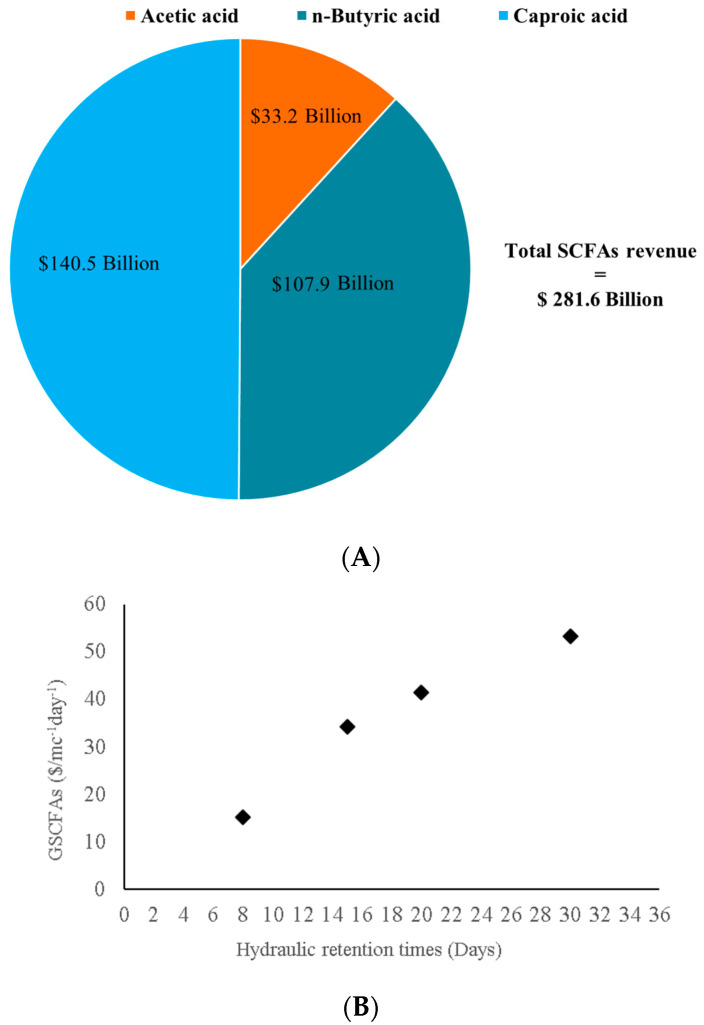
(**A**) Estimation of SCFAs revenue from AF of SBM at HRT of 30 days and 25 °C temperature condition. (**B**) Specific digester productivity at different HRTs.

On the other hand, considering the highest SCFAs concentration reached in the present research, an estimation of the economic revenue from the process performed herein was addressed (Figure 4). Given the daily input of molasses into this reactor, the total SCFAs concentration obtained at HRT 30 days was 13.4 g/g_Molasses_. The prevailing SCFAs in this reactor were acetic, n-butyric, and caproic acids, with percentages of 38.2 ± 2.3%, 27.7 ± 2.1%, and 25.4 ± 2.9%, respectively. SCFAs with low proportions in the fermentation broth were excluded from this estimation. The current SCFAs prices considered were adapted from Agnihotri et al. [45], being $600, $2686, and $3815, per ton of acetic, butyric, and caproic acids, respectively. As can be seen in Figure 4, a total revenue of $281.61 billion can be generated from SCFAs production through AF of beet molasses at 25 °C, which is largely superior to the income from selling molasses as an animal feed supplement. Notwithstanding that, SCFAs extraction/separation from the fermentation broth is a subsequent process to AF to be considered when calculating the costs of the overall process [45,46]. Nevertheless, for this calculation, only the product costs are considered, namely animal feed vs. carboxylates as biochemicals. In this regard, a detailed techno-economic design of a full-scale SBM-AF process should be conducted to assess its real applicability.

The second approach to evaluate the effect of HRTs on the revenues from the AF of SBM in the studied conditions was adopted from Montecchio et al. [47], where the parameter “specific digester productivity” refers to SCFAs revenues and was calculated as GSCFAs = (Q × SCFAs)/Vol reactor. SCFAs are expressed as revenues ($) and GSCFAs is, therefore, defined as $/m^3^ day^−1^. The reactor working volume was 0.001 m^3^, as described in the M&M section. The SCFAs concentrations considered for this calculation (expressed as g/L) were those of the predominant carboxylates in each condition and, their prices were according to Agnihotri et al. [45], as in the previous estimation. As can be seen in Figure 4B, the highest revenue could be reached concomitantly with the HRTs increase (from 8 to 30 days). Therefore, an almost linear relationship could be established between the two parameters.

In future research, other systems allowing shortening HRT while retaining biomass-like sequencing batch reactors (SBR) could also be considered to further optimize the productivity of SCFA from beet molasses at 25 °C. Another possible approach is using hybrid bioreactors, in which the CSTR configuration is modified by including support material inside the reactors to promote microorganisms’ growth and retention (increasing the cellular retention time).

## 3. Materials and Methods

### 3.1. Substrate and Inoculum

SBM (COMPAÑIA DE MELAZAS S.A., Madrid, Spain) was the primary substrate for SCFAs production. The characteristics of SBM are depicted in Table 2. SBM mainly contained sucrose (above 95% of the composition); thus, it was expected to be a suitable raw material for SCFAs production through AF. This substrate was selected based on its high carbohydrate content and since it does not exhibit low hydrolytic efficiencies (as happens with particulate wastes) to produce SCFAs via AF. In this way, by ensuring an optimum hydrolytic stage, this research can be entirely devoted to investigating the acidogenesis stage.

The inoculum was an anaerobic sludge collected from a local wastewater treatment plant (El Soto-Móstoles, Madrid, Spain). The mesophilic (35 °C) anaerobic sludge had the following physicochemical characterization: pH 7.3 ± 0.1; TS = 14.5 ± 1.3 g/L; VS = 8.2 ± 1.9 g/L; N-NH_4_^+^ = 0.8 ± 0.1 g N/L and alkalinity = 4.3 ± 0.1 g CaCO_3_/L.

### 3.2. Experiments Setup

The AF of SBM was carried out in semi-continuous reactors (working volume of 1 L) at 25 °C. The temperature was maintained using a thermostatic water bath (F12-ED v2.0, Julabo, Seelbach, Germany). The mixing was performed by magnetic stirring using agitators (MR Hei-MixL, Heidolph Instruments, Schwabach, Germany). The applied low temperature (25 °C) was based on previous studies on AF for SCFAs production and is economically promising compared to higher temperatures where an energy supply for heating is needed [2,22]. A fixed OLR of 4 g COD/L·d was used, and different HRTs of 30, 20, 15, and 8 days were tested to identify the most suitable. Each reactor was operated for at least three times its HRT and until stable output concentrations were achieved.

Since SBM is a carbohydrate-rich material, the fast hydrolysis and acidification of sugars might lead to a fast pH decrease. To avoid a drastic pH drop that would result in the accumulation of primary metabolites (e.g., ethanol and lactic acid) instead of SCFAs [17], pH was monitored daily and adjusted to slightly acidic values (5.5–6.5) using 5 M of NaOH. This methodology was based on previous studies that identified this pH range as optimal for AF performance [2,17,31].

### 3.3. Analytical Methods

SBM used as feedstock and effluents from CSTRs were analyzed in terms of total solids (TS), volatile solids (VS), and chemical oxygen demand (COD), according to Standard Methods [48]. The samples for analyzing SCFAs, soluble chemical oxygen demand (SCOD), and ammonium (N-NH_4_^+^) were filtered by a 0.45 µm filter membrane. SCOD and N-NH_4_^+^ were analyzed using colorimetric commercial kits (ISO 7150-1, and ISO 15705, respectively. Merck, Darmstadt, Germany). Samples for SCFAs were further filtered by a 0.22 µm Teflon filter and analyzed via liquid chromatography (HPLC) (HPLC-RID 1260, Agilent, Santa Clara, CA, USA) with a refractive index detector. HPLC was equipped with an ion exclusion column (Aminex HPX-87H with 300 × 7.8 mm internal diameters) using the conditions described by Llamas et al. [16]. The analyzed SCFAs were lactic, acetic, propionic, isobutyric, butyric, isovaleric, valeric, and caproic acids. The content of ethanol was also analyzed using the same methodology described for SCFAs. Similarly, sucrose and fructose content in SBM was determined in the HPLC using a CARBOSep CHO-682 column (Transgenomic, Omana, NE, USA) under the conditions detailed by Cubas-Cano et al. [49], after filtering the samples by a 0.22 µm Teflon filter. Total Kjeldahl nitrogen (TKN) was determined according to the method 4500-N of the standard methods [50], and protein content was obtained from the value of the TKN content and applying a conversion factor (i.e., 6.25) [43,49]. Carbohydrates were measured using the phenol-sulfuric acid method [51]. Biogas composition in terms of H_2_, CO_2_, and CH_4_ was determined using gas chromatography according to Greses et al. [2].

### 3.4. Calculations for the Evaluation of Process Performance

SCFAs yield was determined as the net SCFAs concentration (expressed as grams of COD_out_) divided by the total COD of the feedstock (TCOD_in_):

$$SCFAs\;Yield(g/gCODin)=\frac{COD(SCFAs)out}{TCODin}$$

The conversion factors of SCFAs concentrations into COD correspond stoichiometrically to the following values: 1.067 for acetic acid, 1.514 for propionic acid, 1.818 for n-butyric acid (the sum of iso-butyric and butyric), 2.039 for n-valeric (the sum of iso-valeric and valeric), and 2.207 for caproic acid. Thereby, concentrations in g/L were multiplied by each factor to attain g COD/L.

The solubilization and acidification degrees refer to the efficiencies of solubilizing (hydrolysis stage) and acidifying (acidification stage) the organic material fed (COD_in_) to the reactor, respectively. Those percentages can be calculated as follows:

$$\%\;Solubilization\;degree=\frac{SCOD}{TCOD}\times 100$$

$$\%\;Acidification\;degree=\frac{COD(SCFAs)}{SCOD}\times 100$$

COD removal refers to the organic material, in terms of total COD, that has been removed from the system and transformed into one or various end-products other than SCFAs. This percentage can be calculated as follows:

$$\%\;COD\;removal=\frac{\left(CODin-CODout\right)}{CODin}\times 100$$

## 4. Conclusions

The HRT effect on AF of SBM at 25 °C revealed a lack of significant variations on acidogenesis degree and SCFAs yields when this parameter ranged from 8 to 30 days. However, an apparent effect on microbial metabolisms was detected since the SCFAs profile was altered. Long HRTs (i.e., 30 and 20 days) were identified to be the most suitable to obtain long carbon chain carboxylates via CCE since high caproic acid concentrations (15.4 ± 1.5 g COD/L) were reached at the HRT of 30 days. Furthermore, the operation at long HRTs led to a more stable process, which is crucial for scaling up the technology. This investigation provided an efficient strategy to valorize SBM wastes for SCFAs production, which exhibits higher economic revenue than its uses for animal feed preparation. Aiming to decrease society’s dependency on petrochemicals, SCFAs are essential building blocks in different industrial sectors.

In terms of SCFAs yield, the HRT of 8 days showed the highest values in terms on g SCFAs/g COD_in_. However, the HRT of 30 days exhibited the highest stability and allowed the obtaining of a distribution profile rich in caproic acid.

## Figures and Tables

**Table 1:** Characteristics of the effluents from AF operated at different HRT.

Parameters (Units)	HRT 8 d	HRT 15 d	HRT 20 d	HRT 30 d
pH	5.4 ± 0.3 **	5.4 ± 0.2 **	5.6 ± 0.3 *	5.8 ± 0.2 *
TS (g/L)	16.6 ± 3.4	25.2 ± 2.6	37.1 ± 4.6	54.4 ± 4.4
VS (g/L)	10.3 ± 1.7	17.2 ± 2.3	22.6 ± 1.9	34.4 ± 2.6
TCOD (g/L)	24.5 ± 3.1	41.5 ± 4.2	53.2 ± 0.9	70.5 ± 0.8
SCOD/TCOD	0.8 ± 0.1	0.8 ± 0.1	0.9 ± 0.1	0.9 ± 0.5
Total SCFA (g/L)	8.5 ± 0.6	11.5 ± 0.6	20.4 ± 0.5	27.6 ± 1.6
Caproic acid yield (gCOD/gCOD_in_)	0.07 ± 0.01	0.09 ± 0.01	0.13 ± 0.01	0.013 ± 0.010
SCFAs yield gCOD/gVS_in_	0.6 ± 0.1	0.5 ± 0.1	0.5 ± 0.1	0.6 ± 0.1
SCFAs yield gCOD/gCOD_in_	0.43 ± 0.03	0.35 ± 0.02	0.39 ± 0.01	0.37 ± 0.02
COD removal (%)	23.8 ± 9.2	31.8 ± 6.8	37.9 ± 1.1	41.6 ± 0.7
H_2_ yield (ml/gCOD_in_)	12.6 ± 1.2	36.9 ± 5.9	32.6 ± 8.6	55.9 ± 8.9

*: pH control only at the startup; ** pH fluctuations were pronounced, and the control was throughout the experiment.

**Table 2:** Physicochemical characterization of SBM (mean ± SD).

Parameters (Units)	SBM
pH	6.7 ± 0.1
TS (%)	76.4 ± 0.2
VS (%)	66.6 ± 0.9
TCOD (g/L)	1131.0 ± 4.5
SCOD/TCOD	0.9 ± 0.1
TCOD/VS	1.7 ± 0.1
Total sugars (g/L)	695.6 ± 1.8
Sucrose (g/L)	665.6 ± 0.4
Glucose (g/L)	30.0 ± 1.4
N-NH_4_^+^ (g N/L)	0.5 ± 0.1
Alkalinity (g CaCO_3_/L)	1.7 ± 0.2
Carbohydrates (%) *	56.6 ± 0.8
Ash (%) *	9.8 ± 0.6
Lipids (%) *	20.7 ± 0.2
Proteins (%) *	12.9 ± 0.3

* Percentage calculated based on dry matter content. TS: Total solids; VS: Volatile solids; N-NH_4_^+^: Ammonium nitrogen; TCOD: Total chemical oxygen demand; SCOD: Soluble chemical oxygen demand.

## Data Availability

Not applicable.

## Supplementary Materials

The following supporting information can be downloaded at: https://www.mdpi.com/article/10.3390/molecules28186635/s1, Figure S1: pH evolution throughout the experiment at the different HRTs. Red lines refer to the upper and lower suitable values for anaerobic fermentation aiming to produce SCFAs.
