# Supplementary material for: Hydraulic Retention Time as an Operational Tool for the Production of Short-Chain Carboxylates via Anaerobic Fermentation of Carbohydrate-Rich Waste
Source: Molecules. 2023 Sep 15;28(18):6635. doi: 10.3390/molecules28186635 (PMC10537262; doi:10.3390/molecules28186635)
Supplement: Supplementary file 1 [file molecules-28-06635-s001.zip › molecules-2548517-supplementary.pdf]

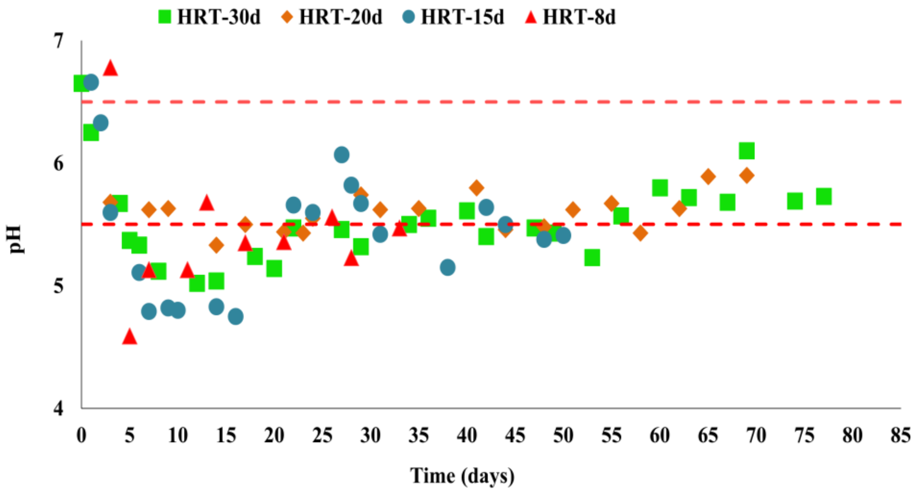

Figure S1. pH evolution throughout the experiment at the different HRTs. Red lines refer to the upper and lower suitable values for anaerobic fermentation aiming to produce SCFAs.
